# LB9. Longitudinal antibody dynamics in children infected with SARS-CoV-2 through 6 months post-infection

**DOI:** 10.1093/ofid/ofab466.1640

**Published:** 2021-12-04

**Authors:** Lauren E Gentles, Leanne P Kehoe, Katharine D Crawford, Kirsten Lacombe, Jane Dickerson, Joanna H Yuan, Susanna L Schuler, Sankan Nyanseor, Sharon Saydah, Claire Midgley, Kimberly Pringle, Jesse Bloom, Janet A Englund

**Affiliations:** 1 Fred Hutchinson Cancer Research Center, Seattle, WA; 2 Seattle Children’s Hospital, Seattle, Washington; 3 University of Washington, Seattle, Washington; 5 U.S. Centers for Disease Control and Prevention, Atlanta, Georgia; 6 Centers for Disease Control and Prevention, Atlanta, Georgia; 8 Seattle Children’s Hospital/Univ. of Washington, Seattle, Washington

## Abstract

**Background:**

Severe acute respiratory syndrome coronavirus 2 (SARS-CoV-2) infection elicits antibodies (Abs) that bind several viral proteins such as the spike entry protein and the abundant nucleocapsid (N) protein. We examined convalescent sera collected through 6 months (~24wks) post-SARS-CoV-2 infection in children to evaluate changes in neutralization potency and N-binding.

**Methods:**

Outpatient, hospitalized, and community recruited volunteers < 18 years with COVID-19 were enrolled in a longitudinal study at Seattle Children’s Hospital. Analysis includes symptomatic and asymptomatic children with laboratory-confirmed SARS-CoV-2 infection who provided blood samples at approximately 4wks (range: 2-18wks, IQR:4-8wks) and 24 wks (range: 23-35wks, IQR:25-27wks) after diagnosis. We measured neutralizing Ab using an in-house pseudoneutralization assay and anti-N binding Ab using the Abbott Architect assay.

**Results:**

Of 32 children enrolled between April 2020 and January 2021, 27 had no underlying immunocompromised state and 25 of these 27 children had symptomatic disease. Ten of 27 had a > 2-fold decrease neutralization titers between 4 and 24wks (most were < 10-fold); 12 had < 2-fold change; and 5 had neutralization titers that increased > 2-fold over time (Fig. 1A). All but one of these 27 children had detectable neutralizing activity at 24wks. Anti-N Abs were assessed for 25 children at 4wks and 17 children at 24wks (data pending for 14 samples); all children with paired samples had a > 1.75-fold Abbott index reduction at 24wks, and 5 children had no detectable anti-N Abs by 24wks (Fig. 2A). An additional 5 children with symptomatic disease had complicating immunosuppression or multiple blood transfusions; 2 had decreasing neutralizing titers, 2 increased, and 1 had no change (Fig. 1B). Anti-N Abs were undetectable for one child by 24wks (data pending for 4 samples) (Fig. 2B). No participants received COVID-19 vaccine.

Figure 1. Pseusoneutralization titers in children over time.

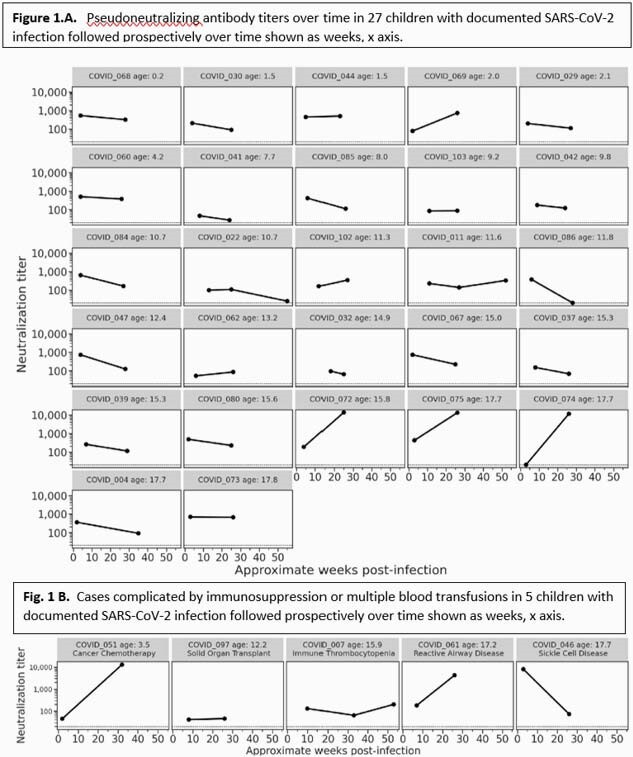

Figure 2. Nucleocapsid-binding antibody titers in children over time.

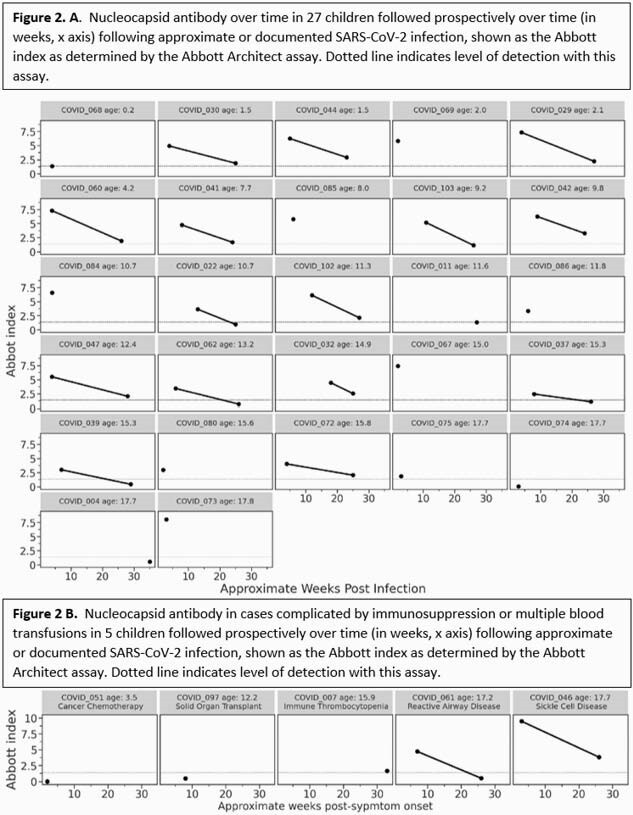

**Conclusion:**

We show neutralizing Abs wane to a small degree over 24wks post-SARS-CoV-2 infection and remain detectable in most children. In contrast, anti-N Abs decreased, becoming undetectable in some children by 24wks. These findings add to understanding of the natural history of SARS-CoV-2 immunity in children.

* This study was supported by CDC BAA75D301-20-R-67897

**Disclosures:**

**Jesse Bloom, PhD**, **Flagship Labs 77** (Consultant)**Moderna** (Consultant) **Janet A. Englund, MD**, **AstraZeneca** (Consultant, Grant/Research Support)**GlaxoSmithKline** (Research Grant or Support)**Meissa Vaccines** (Consultant)**Pfizer** (Research Grant or Support)**Sanofi Pasteur** (Consultant)**Teva Pharmaceuticals** (Consultant)

